# ﻿A new species of the rare genus *Endogeophilus* from southern France, with a key to the European genera of Geophilidae s.l. (Chilopoda)

**DOI:** 10.3897/zookeys.1213.133171

**Published:** 2024-09-26

**Authors:** Étienne Iorio, Lucio Bonato

**Affiliations:** 1 EI – Entomologie & Myriapodologie, 36 impasse des Acacias, F-84260 Sarrians, France EI – Entomologie & Myriapodologie Sarrians France; 2 Dipartimento di Biologia, Università di Padova, via U. Bassi 58b, I-35131 Padova, Italy Università di Padova Padova Italy; 3 National Biodiversity Future Centre, Palermo, Italy National Biodiversity Future Centre Palermo Italy

**Keywords:** Disjunct distribution, endogeic, Europe, Geophilomorpha, morphology, Provence, Sardinia

## Abstract

The geophilid centipede *Endogeophilusalberti***sp. nov.** is described and illustrated based on a single specimen collected from Provence, southern France. It is very similar to *E.ichnusae* Bonato, Zapparoli, Drago & Minelli, 2016, which is known only from three specimens from south-western Sardinia, and was the only species in the genus *Endogeophilus* Bonato, Zapparoli, Drago & Minelli, 2016. Both species share a remarkably narrow body, very short setae, and an unusually high number of legs, which are relatively stout. All these traits are rare among geophilids and suggest an endogeic life style. Despite of the very few specimens available for comparison and the difficulties to distinguish inter-specific differences from intra-specific variation, the two species differ at least in the shape of the pretarsi of the second maxillae and the shape of the forcipules. A revised diagnosis of the genus *Endogeophilus* is also provided, with an identification key to all genera of Geophilidae s.l. recorded so far in Europe, based on selected characters to evaluate without anatomical dissection and illustrated with original pictures.

## ﻿Introduction

*Endogeophilus* Bonato, Zapparoli, Drago & Minelli, 2016 is a peculiar lineage of European geophilids, showing morphological traits that suggest strictly endogeic life (Bonato et al. 2016). However, it is one of the most infrequently found geophilids, despite the European soil biotas have been intensely sampled in the past: up to date, only three specimens have been reported, from two sites in south-western Sardinia, and they have been recognized as conspecific (*Endogeophilusichnusae* Bonato, Zapparoli, Drago & Minelli, 2016).

**Figure 1.**
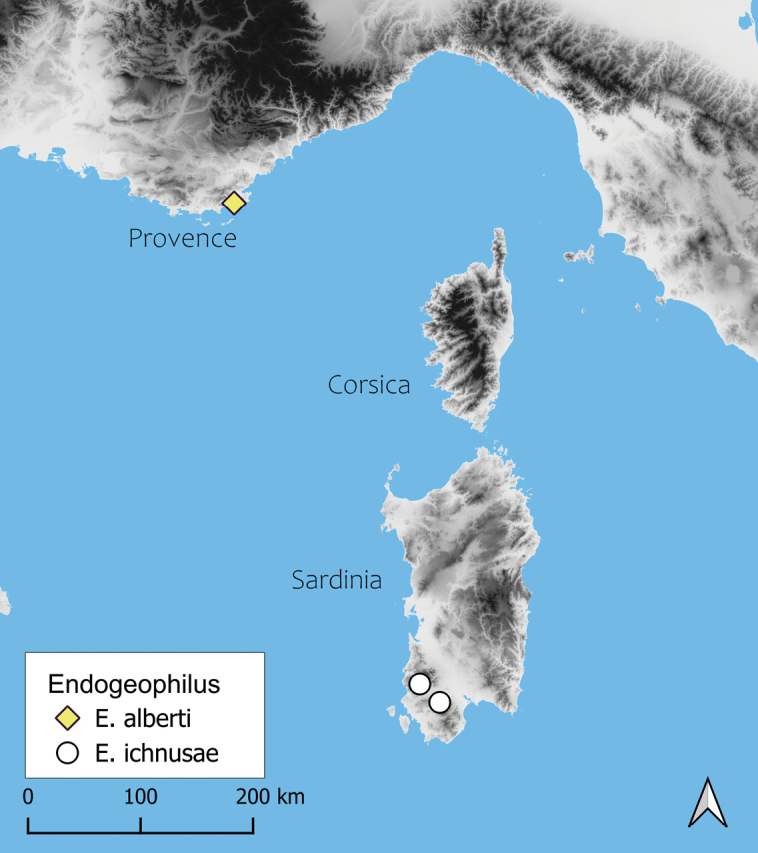
Distribution of all known records of *Endogeophilus*.

Here we report on a new record of *Endogeophilus*, the first from continental Europe, precisely from Provence, southern France (Fig. 1). Only a single specimen has been found; nevertheless the critical examination of its morphological features indicates that it represents a new species, clearly related to *Endogeophilusichnusae*.

Besides describing *Endogeophilusalberti* sp. nov. and discussing differences between the two species of *Endogeophilus*, we also provide a revised diagnosis of the genus and an identification key to assist in distinguishing it from all other European geophilids.

## ﻿Materials and methods

A total of 758 centipedes (Chilopoda) were collected by the first author in the Port-Cros National Park (both in the central and the peripheral area, from Ramatuelle to Hyères, islands included). They were collected mainly by hand and by soil and litter sieving, in several sessions between 2019 and 2024. All specimens have been examined by the first author, with a Motic SMZ168 T-LED stereo microscope and a Motic Elite B1-223E-SP trinocular microscope. Geophilids have been identified at the species level. For the taxonomy and nomenclature, we followed Bonato and Minelli (2014), Iorio (2014), and subsequent papers (Bonato et al. 2016, 2023). For the diagnostic characters, we followed Brolemann (1930) and Bonato et al. (2014a).

Only one specimen of the new species was found, despite several further attempts aimed at collecting other specimens. The single specimen was compared to representatives of other known geophilid species, including a paratype of *Endogeophilusichnusae* (in the collection of the University of Padova, code PD 1373; see Bonato et al. 2016).

Measurements were taken with two micrometres applied to the B1-223E-SP microscope, with precision 0.1 mm or 0.01 mm. Photos were taken with a Moticam 5 camera applied to the same microscope and stacked with Helicon Focus 8.2.2. For describing the morphology, we followed the terminology recommended by Bonato et al. (2010). Abbreviation: **LBS** = leg-bearing segment(s).

In order to facilitate the distinction of specimens of *Endogeophilus* from similar centipedes, and to provide a practical tool for sorting samples of European geophilids, we produced an original identification key to all genera of Geophilidae known from Europe. We considered the Geophilidae sensu lato (i.e., comprising genera previously separated in other families, like Dignathodontidae Cook, 1896 and Linotaeniidae Cook, 1899, to encompass a probably monophyletic group; see Bonato et al. 2014b) and we followed the conventional geographic boundaries adopted by Fauna Europaea (de Jong 2016) and by recent synopses on European Geophilomorpha (Bonato and Minelli 2014; Bonato et al. 2014a). The key was based on both published information on morphology (Brolemann 1930; Verhoeff 1941; Machado 1952; Minelli 1982; Lewis et al. 1988; Bonato et al. 2006, 2011, 2012a, 2012b, 2014a, 2016; Bonato and Minelli 2008; Barber 2009; Tuf and Dányi 2015; Iorio 2016; Dányi and Tuf 2017; Iorio and Quindroit 2018; Iorio et al. 2022b; Desmots and Racine 2023; Dyachkov and Bonato 2024) and original observations on specimens representative of different genera. Priority was given to characters that are effective for both adult and immature specimens, easier to evaluate and less prone to subjective interpretation or misinterpretation.

Photographs illustrating the key were taken of the following specimens:

- *Acanthogeophilusspiniger* (Meinert,1870): 1 ♀, Edough Massif (Algeria), 23.X.1984, leg. unknown, det. L. Bonato, PD-G 5153
- *Arctogeophilusinopinatus* (Ribaut, 1911): 2 ♂, 2 ♀, Mervent (Vendée, France), forest of Mervent, old oak forest, 05.V.2015, leg./det. É. Iorio
- *Arenophilusperegrinus* Jones, 1989: 1 ♀, Noirmoutier-en-l’Île (Vendée, France), les Cents, leg. D. Desmots, det. A. Racine. Specimen reported by Desmots and Racine (2023)
- *Clinopodesvesubiensis* Bonato, Iorio & Minelli, 2011: ♀, Lucéram (Alpes-Maritimes, France), Peira-Cava, La Cabanette, 1320–1450 m a.s.l., mixed forest, 09.III.2007, leg. É. Iorio, det. L. Bonato and É. Iorio
- *Dignathodonmicrocephalus* (Lucas, 1846): ♀, Roquebillière (Alpes-Maritimes, France), Berthemont, 950 m a.s.l., deciduous forest, 07.VII.2007, leg./det. É. Iorio
- *Eurygeophiluspinguis* (Brolemann, 1898): ♀, Loubens (Ariège, France), cave of Portel, 16.III.2014, leg. O. Courtin, det. É. Iorio
- *Galliophilusbeatensis* Ribaut & Brolemann, 1927: ♂, Escouloubre (Aude, France), forest of Carcanet, 04.VI.2015, leg. H. Brustel, det. A. Racine and É. Iorio
- *Geophiluselectricus* (Linnaeus, 1758): ♂♀, la Chapelle-en-Valgaudémar (Hautes-Alpes, France), 44.8179°N, 6.1816°E (WGS84), 1146 m a.s.l., forest edge, 04.V.2021, leg. F. Noël, det. É. Iorio
- *G.flavus* (De Geer, 1778): ♂♀, la Chapelle-en-Valgaudémar (Hautes-Alpes, France), 44.8179°N, 6.1816°E (WGS84), 1146 m a.s.l., forest edge, 04.V.2021, leg. F. Noël, det. É. Iorio
- *G.fucorum* Brolemann, 1909: ♂♀, Hyères (Var, France), Port-Cros island, sandy beach of Port-Man, 43.0100°N, 6.4113°E (WGS84), west side with a thick stranded *Posidonia* “banquette”, 04.IV.2019, leg./det. É. Iorio
- *G.gavoyi* Chalande, 1910: ♂, Mandelieu (Alpes-Maritimes, France), mixed forest, 07.V.2010, leg./det. É. Iorio
- *G.osquidatum* Brolemann, 1909: ♂♀, Torcé-Viviers-en-Charnie (Mayenne, France), forest of the Grande Charnie, 48.0693°N, -0.2591°E (WGS84), oak forest with temporary stream, 05.IV.2016, leg./det. É. Iorio
- *G.richardi* Brolemann, 1904: 1 ♂, 2 ♀, Sospel (Alpes-Maritimes, France), river of the Bévéra, riparian forest, 26.III.2018, leg. J.-M. Lemaire, det. É. Iorio
- *G.studeri* Rothenbühler, 1899: ♀, la Chapelle-en-Valgaudémar (Hautes-Alpes, France), 44.8243°N, 6.2625°E (WGS84), 1386 m a.s.l., beech forest, 03.V.2021, leg. F. Noël, det. É. Iorio
- *Gnathoribautiabonensis* (Meinert,1870): ♀, Colares (Lisbon district, Portugal), 38.8302°N, -9.4686°E, on a cliff, 28.XII.2018, leg. T. Cherpitel and M. Filipe, det. A. Racine and É. Iorio
- *Heniabicarinata* (Meinert, 1870): ♀, La Croix-Valmer (Var, France), Cap Taillat, 43.1714°N, 6.6414°E, gravel beach with a stranded Posidonia “banquette”, 24.III.2022, leg./det. É. Iorio
- *H.brevis* (Silvestri, 1896): ♀, Sospel (Alpes-Maritimes, France), 43.9042°N, 7.4481°E, 620 m a.s.l., oak forest, in a shaded valley 05.XI.2020, leg./det. É. Iorio
- *H.vesuviana* (Newport, 1844): ♂♀, Metz (Moselle, France), Fort of Queuleu, deciduous forest in old fortifications, 01.V.2002, leg./det. É. Iorio
- *Pachymeriumferrugineum* (C.L. Koch, 1835): 1 ♂, 2 ♀, Hyères (Var, France), Giens, beach of l’Ayguade, 43.0402°N, 6.0964°E (WGS84), under stones and stranded *Posidonia*, 26.X.2015, leg. F. Noël, det. É. Iorio
- *Pleurogeophilusmediterraneus* (Meinert, 1870): ♂♀, Sospel (Alpes-Maritimes, France), 43.8426°N, 7.4477°E (WGS84), 729 m a.s.l., mixed forest, northern slope, 10.XI.2020, leg./det. É. Iorio
- *Stenotaenialinearis* (C.L. Koch, 1835): 2 ♀, Ramatuelle (Var, France), Moulin of Paillas, 43.2135°N, 6.6070°E, 250 m a.s.l., old oak forest, 13.II.2024, leg./det. É. Iorio
- *Strigamiacarniolensis* (Verhoeff, 1895): 3 ♀, Chantepérier (Isère, France), 45.0123°N, 5.9757°E, 1442 m a.s.l., small beech forest, 10.V.2021, leg. F. Noël, det. É. Iorio
- *Tuobaposeidonis* (Verhoeff, 1901): 7 ♂, 11 ♀, Hyères (Var, France), Port-Cros island, “Fond de la Rade”, 43.0068°N, 6.3820°E, well preserved gravel beach with stranded *Posidonia*, 05.IV.2019, leg./det. É. Iorio.

All specimens are in the collection of ÉI, with the exception of *Acanthogeophilusspiniger*, which is in the Minelli-Bonato collection, and *Arenophilusperegrinus*, which is in the collection of Antoine Racine. All photos were taken by ÉI, with the exception of those of *Acanthogeophilusspiniger*, taken by LB, and of *Arenophilusperegrinus*, taken by A. Racine.

## ﻿Results

### ﻿ Endogeophilus

Taxon classificationAnimaliaGeophilomorphaGeophilidae

Bonato, Zapparoli, Drago & Minelli, 2016

B717CD77-3895-5C92-8184-2DB9CDADC377

#### Diagnosis.

Geophilids with the following combination of characters: body remarkably narrow (length/width ratio ~ 70); setae relatively short (not surpassing 30 μm on the head); head slightly longer than wide; clypeus uniformly areolate; labrum with tubercles on the intermediate part and bristles on the side-pieces, which are distinct from the clypeus; second maxillary coxosternite with a long isthmus, without inner processes and without sclerotized ridges; pretarsus of second maxillae claw-like; forcipular tergite relatively broad (posterior margin about as wide as the subsequent tergite); forcipular coxosternite without anterior denticles, with complete coxopleural sutures distinctly diverging anteriorly, and with complete chitin-lines; forcipule with a single denticle, relatively small, on the tarsungulum; metasternites of the anterior part of the trunk with carpophagus pits and with pore-fields, a sub-ovoid/sub-triangular pore-field (approximately as long as wide or slightly longer than wide) on the posterior part of each metasternite; metasternites slightly longer than wide at ~ 20% of the series of trunk segments; > 90 pairs of legs, all relatively short (length/width ratio of leg tarsi < 2.5) and with slender accessory spines; metasternite of the ultimate leg-bearing segment trapezoid, wider than long; coxal organs opening through separate pores, most of them close to the metasternite, one isolated on the ventral side of the coxopleuron and some on the dorsal side; legs of the ultimate pair distinctly longer than the penultimate legs, with a claw-like pretarsus.

#### Type species.

*Endogeophilusichnusae* Bonato, Zapparoli, Drago & Minelli, 2016, by original designation.

### ﻿ Endogeophilus alberti sp. nov.

Taxon classificationAnimaliaGeophilomorphaGeophilidae

8BE83622-70C5-5EB4-B876-324E5316EA3F

https://zoobank.org/D21886AF-5DFE-4248-8532-8D84E673C6DA

#### Type specimen.

**Figure 2.**
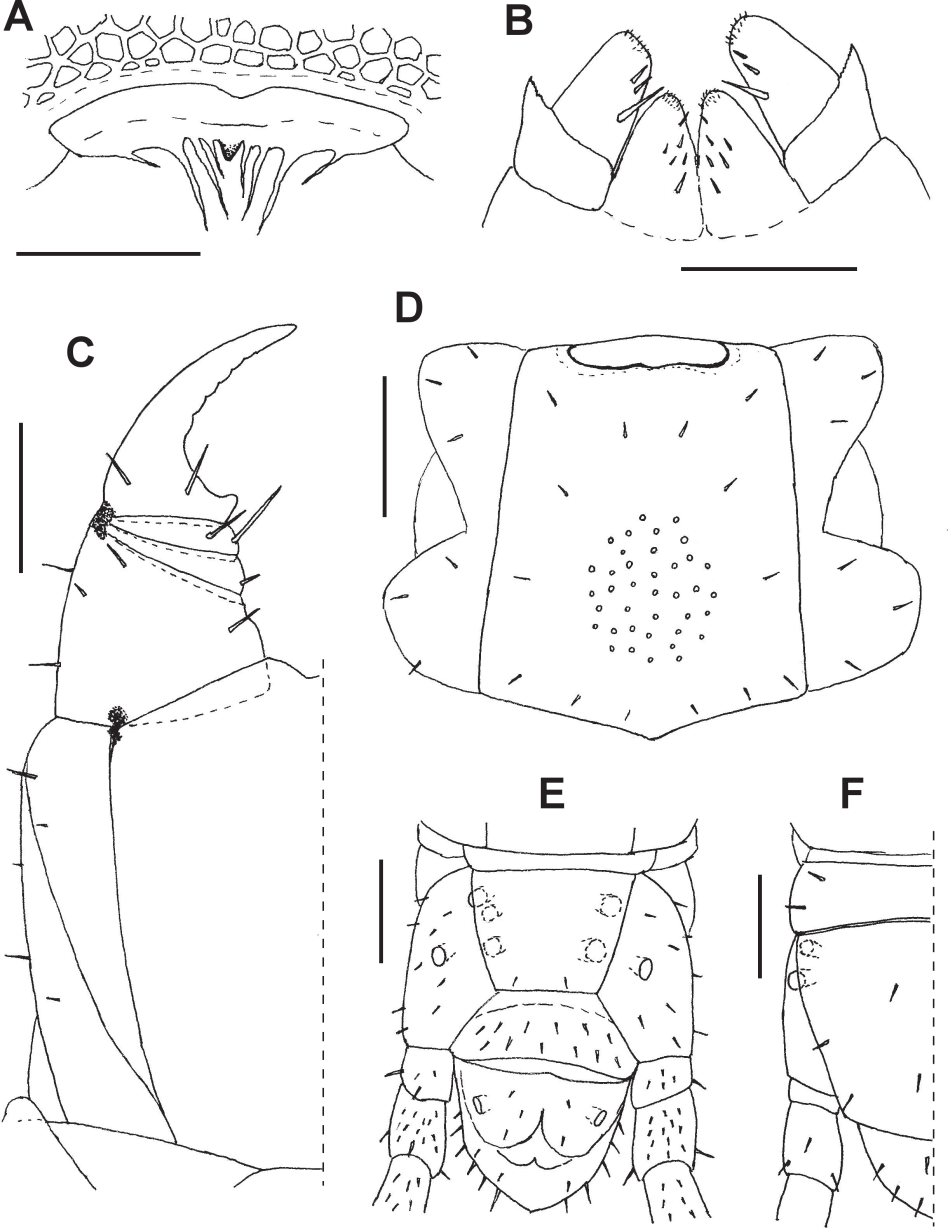
*Endogeophilusalberti* sp. nov., holotype **A** labrum **B** first maxillae **C** right part of the forcipular segment **D**LBS 18 **E** ultimate LBS without distal part of legs **F** left part of the ultimate LBS without distal part of leg. Views: ventral (**A–E**), dorsal (**F**). Scale bars: 20 µm (**A**); 40 µm (**B**); 100 µm (**C–F**).

**Figure 3.**
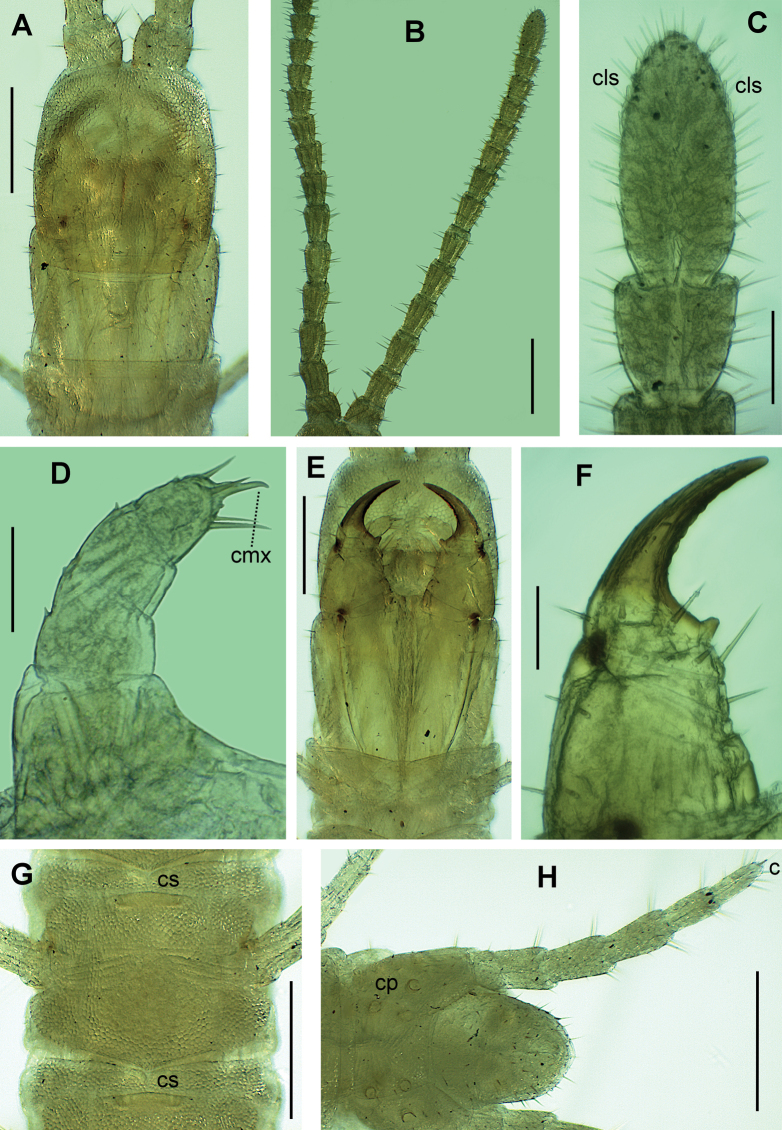
*Endogeophilusalberti* sp. nov., holotype **A** head and forcipular segment **B** antennae **C** distal articles of left antenna **D** right telopodite of second maxillae **E** head and forcipular segment **F** right forcipule **G**LBS 15 and anterior part of 16 **H** ultimate LBS without right leg. Views: ventral (**D–H**), dorsal (**A–C**). Abbreviations: cls club-like sensilla, cmx claw of the second maxillae, cp coxopleural pores, cs carpophagus-structure. Scale bars: 200 µm (**A–B, E, G–H**); 50 µm (**C–D, F**).

***Holotype*** (Figs 2, 3): • ♀, 31.III.2023, É. Iorio leg. In ethanol. Body in four pieces: head, maxillae, mandibles, and trunk. Original label: Cavalaire-sur-Mer (Var), Malatra, “MALAT3”, 43.1795°N, 6.5068°E (WGS84), 31.III.2023, É. Iorio leg. Deposited in the Muséum national d’Histoire naturelle, Paris (Chilopoda collection, number M370).

#### Type locality.

France: Var department: Cavalaire-sur-Mer: near Malatra: 43.1795°N, 6.5068°E (WGS84), 215 m a.s.l., north-eastern slope (Fig. 1).

#### Diagnosis.

**Figure 4.**
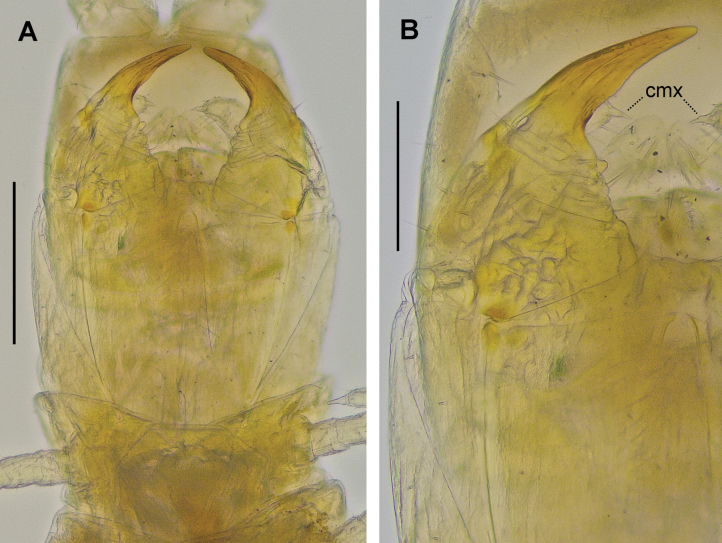
*Endogeophilusichnusae*, paratype PD 1373, ventral **A** head and forcipular segment **B** right forcipule and apical claw of the second maxillae. Abbreviation: cmx claw of the second maxillae. Scale bars: 100 µm.

An *Endogeophilus* species with claw of the second maxillae slender and hooked at its tip; forcipular trochanteroprefemur ~ 1.1× as long as wide; forcipular tarsungulum > 2.0× as long as wide, almost as long as the trochanteroprefemur, distinctly curved, fairly slender, and gradually narrowing. See also Table 1, Figs 2–4, and Discussion.

**Table 1.** Differences between the single specimen of *Endogeophilusalberti* sp. nov. and the three specimens of *E.ichnusae*. For each character, each possible explanation (intraspecific variability, errors in character evaluation or divergence between species) has been evaluated as probable (x), possible (?) or improbable (–), based on what is known from other Geophilidae. Data are from Bonato et al. (2016) and this study.

Species	*E.alberti* sp. nov.	*E.ichnusae*	Explanations				
Specimens examined	1, female, 24.5 mm	3, both sexes, 31–34 mm	Differences in body size	Differences between sexes	Variability between individuals	Errors in character evaluation	Differences between species
Head: setae: max length	22 μm	15 μm	–	–	?	x	?
Labrum: distinction between side-pieces and intermediate part	faint	distinct	x	–	?	?	–
Labrum: number of tubercles	1	2	?	–	x	–	–
Antenna: length/width	3.2	3.6	?	–	x	?	–
First maxillae: coxosternal lappet	absent	small	?	–	x	–	?
First maxillae: telopodital lappet	large	small	?	–	x	–	?
Second maxillae: pretarsus	slender, hooked at the tip	stout, not hooked	–	–	–	–	x
Forcipular metatergite: width/length	2.3	1.8	?	–	x	?	?
Forcipular trochanteroprefemur: length/width	1.1	1.3	–	–	–	–	x
Forcipular tarsungulum: length/width	2.2	1.9	–	–	–	–	x
Forcipule/coxosternite length	0.9–1.0	0.7–0.8	–	–	–	–	x
Forcipular tarsungulum: shape	narrowing gradually	narrowing abruptly	–	–	–	–	x
Anterior part of trunk: metasternite: carpophagus pit	distinct, fairly deep	faint, shallow	?	–	x	–	?
Legs in female: number	99	107	–	–	x	–	?
Coxopleuron: pores: number	5 or 6	9–15	x	–	–	–	?
Ultimate/penultimate telopodite length	1.7	2.0	x	–	–	?	?

#### Description of the holotype

(Figs 2, 3). ***General features*.** Body remarkably slender, 24.5 mm long, uniformly ~ 0.3 mm wide for most part of the trunk, only very slightly narrowing anteriorly along ~ 20 most anterior leg-bearing segments and backwards along approximately the five most posterior leg-bearing segments. Legs relatively short. Colour almost uniformly pale yellow, only the forcipules and the head slightly darker, both pale orange.

***Cephalic capsule and antennae*.** Head 0.4 mm long, sub-rectangular, 1.15× longer than wide (Fig. 3A); the anterior margin slightly angulated with a small medial notch. Transverse suture absent. Approximately 40 setae on the head, the majority being short except those of the lateral margins, longer, reaching up to ~ 22 μm. Clypeus uniformly areolate, without clypeal areas and without plagulae, with six setae, arranged in a longitudinal series of three pairs on the anterior median part of the clypeus. Pleurites uniformly areolate, with some setae. Labrum composed of an intermediate part narrow and almost negligible, having one tubercle (Fig. 2A); side pieces not clearly distinct from the intermediate part of labrum, each with four bristles. Antennae with 14 articles, 1.3 mm long, 3.2× longer than the head (Fig. 3B). article I ~ 0.7× longer than wide, articles II–VII up to 1.7× longer than wide, article VIII ~ 1.2× longer than wide, articles IX–XIII approximately as long as wide; setae gradually denser and shorter from basal to distal articles, both ventrally and dorsally. Article XIV 2.0× longer than wide, with numerous setae and with club-like sensilla grouped on the distal parts of both internal and external sides (Fig. 3C).

***Mandibles and maxillae*.** A single pectinate lamella on each mandible. Coxosternite of the first maxillae entire, without mid-longitudinal sulcus. Coxal projection sub-triangular, longer than wide, bearing one or two basal setae and some more distal spine-like sensilla. Telopodite comprises two articles, the basal one without setae, the distal one with three setae and five or six spine-like sensilla. No coxosternal lappets; telopodital lappets present and pointed (Fig. 2B). Coxosternite of the second maxillae entire, the intermediate part uniformly sclerotised as the remaining parts, its anterior margin concave; no sclerotised ridges. Telopodite of the second maxillae composed of three articles: article 1 with two or three very short external setae, article 2 with one or two very short external setae, article 3 with five or six long distal setae; a simple long apical claw, subconical but hook-shaped at its extremity, with a dorsal bulge at its mid length (Fig. 3D).

***Forcipular segment*.** Tergite trapezoid, the lateral margins distinctly converging anteriorly, ~ 2.3× wider than long, posteriorly almost as wide as the subsequent metatergite (Fig. 3A). Exposed part of the coxosternite ~ 1.1× wider than long; anterior margin with a medial shallow concavity, without denticles. Coxopleural sutures complete, entirely ventral or almost, only very slightly sinuous, and strongly converging posteriorly. Chitin-lines well distinct, reaching the condyles, moderately curved and converging posteriorly (Figs 2C, 3E). Trochanteroprefemur ~ 1.1× as wide as long, the external side ~ 2.2× longer than the internal side, without denticles and with some setae (Figs 2C, 3F). Forcipular intermediate articles distinct, without denticles, each with some long setae. Tarsungulum curved, gradually narrowing, ~ 2.2× as long as wide and with a basal sub-conic tubercle; slightly crenulated in its concavity, with 3–6 shallow projections, less pronounced on the left than on the right (Figs 2C, 3F).

***Leg-bearing segments*.** 99 LBS. No paratergites. Metatergite 1 wider than the subsequent one, lateral margins converging posteriorly, without pretergite. Metatergites with two paramedian sulci. Metasternites slightly longer than wide (length/width ratio ~ 1.1 at ~ 20% of the LBS), uniformly areolate; setae very sparse. Metasternites of LBS 10–20 with a carpophagus pit on the anterior margin, fairly deep on LBS 14–19, ~ 0.6× as wide as the margin of metasternite (Figs 2D, 3G). Glandular pores on the metasternites from the 2^nd^ to the penultimate, up to 30–35 pores and loosely arranged in a sub-ovoid/sub-triangular pore-field on the posterior half of each metasternite in the anterior part of the trunk (Fig. 2D); the pore-fields being much less visible in the posterior half of the trunk, loosely arranged in a transverse band of pores in the ~ 5–10 penultimate LBS. Length/width ratio of leg tarsus ~ 2.3 at ~ 20% of the LBS. Leg claws simple.

***Ultimate leg-bearing segment*.** Setae uniformly sparse. Pleuropretergite entire, lacking sutures or sulci. Metatergite sub-trapezoid, ~ 1.5× wider than long, lateral margins very slightly convex and distinctly converging posteriorly, posterior margin convex. Presternite not medially constricted. Metasternite trapezoid, ~ 1.3× wider than long, anteriorly 1.4× wider than posteriorly (Fig. 2E); lateral margins slightly convex, converging backwards; posterior margin straight. Coxal organs of each coxopleuron opening through five or six independent pores: two dorsal pores, one covered by the pleuropretergite and one exposed on the dorso-lateral side (Fig. 2F); three or four ventral pores including two or three more or less aligned under the edge of the corresponding metasternite and a single pore isolated on the postero-central part of the ventral side (Figs 2E, 3H). Telopodite 0.43 mm long, 7.8× as long as wide, ~ 1.7× as long as the penultimate telopodite; six articles, all covered dorsally with sparse long setae, ventrally with dense shorter setae; a fairly well-developed apical claw (Fig. 3H).

***Postpedal segments*.** Gonopods in the shape of a short, slightly bilobate lamina. A pair of anal pores.

#### Distribution and ecology.

**Figure 5.**
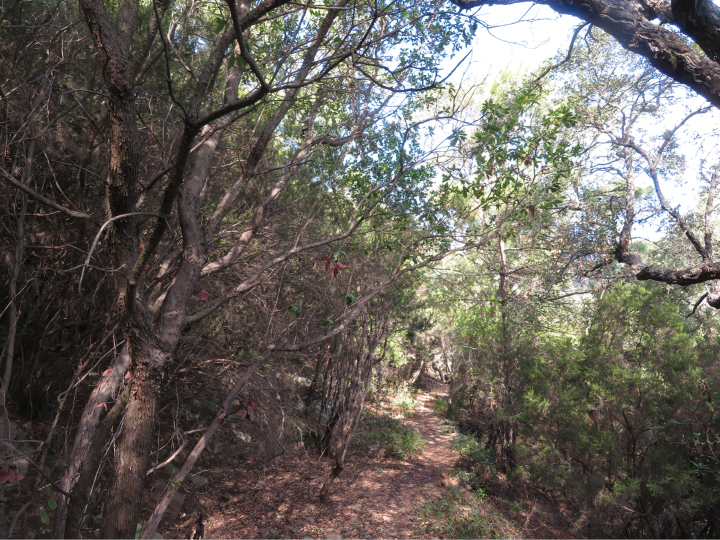
Habitat of *Endogeophilusalberti* sp. nov., near Malatra, Cavalaire-sur-Mer, France (Fig. 1).

The new species is only known from the type locality (see above; Fig. 1). The single specimen was collected in a shaded maquis of old *Arbutusunedo* L., 1753 with also some large *Quercussuber* L., 1753 (Fig. 5). It was found after sieving with a Winkler apparatus, at a depth of 10–20 cm in the soil.

#### Etymology.

This species is dedicated to Prince Albert II de Monaco, because the Foundation Prince Albert II de Monaco has supported the field work of this study. The epithet *alberti* is intended as a noun in the genitive case.

##### ﻿Identification key to the European genera of Geophilidae s.l.

Within Europe, the Geophilidae s.l. can be distinguished from all other Chilopoda by means of the combination of the following two characters: > 25 pairs of legs and second maxillary pretarsi in shape of either subconical, non-spatulate, pointed claw or a tubercle with only one or two tiny spines. A total of 20 genera of Geophilidae s.l. are recorded in Europe (Table 2).

**Table 2.** Genera of Geophilidae s.l. and approximate number of species recognised in Europe. Only genera recorded within the geographic boundaries adopted by Fauna Europaea are considered (see Materials and methods). Occurrences are also indicated for the biogeographical subregions of SW Europe and the two countries where *Endogeophilus* occurs.

Genus	Approximate number of species in Europe	Occurrence		
		South-western Europe	France	Italy
*Acanthogeophilus* Minelli, 1982	1	x	–	x
*Algerophilus* Brolemann, 1925	1	x	–	–
*Arctogeophilus* Attems, 1909	3	–	x	–
*Arenophilus* Chamberlin, 1912	1	x	x	–
*Bebekium* Verhoeff, 1941	1	–	–	–
*Clinopodes* C.L. Koch, 1847	9	x	x	x
*Dignathodon* Meinert, 1870	2	x	x	x
*Diphyonyx* Bonato, Zapparoli & Minelli, 2008	2	–	–	–
*Endogeophilus* Bonato, Zapparoli, Drago & Minelli, 2016	2	x	x	x
*Eurygeophilus* Verhoeff, 1899	2	x	x	x
*Galliophilus* Ribaut & Brolemann, 1927	1	x	x	–
*Geophilus* Leach, 1814	49	x	x	x
*Gnathoribautia* Brolemann, 1909	2	x	–	x
*Henia* C.L. Koch, 1847	17	x	x	x
*Nothogeophilus* Lewis, Jones & Keay, 1988	1	–	–	–
*Pachymerium* C.L. Koch, 1847	6	x	x	x
*Pleurogeophilus* Verhoeff, 1901	3	x	x	x
*Stenotaenia* C.L. Koch, 1847	9	x	x	x
*Strigamia* Gray, 1843	7	x	x	x
*Tuoba* Chamberlin, 1920	2	x	x	x
Total	121 species	16 genera	14 genera	13 genera

The key should be applied by examining any specimen through a microscope. A magnification of 50× is recommended, even 100× for examining labrum and pore-fields. For characters defined on the leg-bearing segments, examination between the 5^th^ and the 20^th^LBS is recommended.

In addition to the dichotomic characters included in the key, additional information is given for each genus (number of species recorded in Europe, geographical distribution within Europe, and additional morphological characters), between square brackets, to assist in the identification.

**Table:** 

1	Legs of the ultimate pair with an apical tubercle having minute spines (Fig. 10B: t)	***Arenophilus* Chamberlin, 1912**
	[In Europe: a single species, *A.peregrinus* Jones, 1989; recorded in very few localities on and near the coasts of south-western England, western France, and western Iberian peninsula. Always with 45 LBS.]	
–	Legs of the ultimate pair with an apical claw or missing the pretarsus at all (Figs 3H, 10A, C–I)	**2**
2	Forcipular tergite sub-rectangular, more or less headband-shaped (Fig. 6D, E: ft)	**3**
	[The genera included here have a large basal denticle on the tarsungulum (Fig. 6F) or a deep median diastema on the forcipular coxosternite (Fig. 6G, H).]	
–	Forcipular tergite trapezoid, posteriorly wider than anteriorly (Fig. 6A–C: ft)	**5**
	[With the exception of *Gnathoribautia* (Fig. 6I), the basal denticle of the tarsungulum, when existing, is smaller (Fig. 7C, G). No deep median diastema on the forcipular coxosternite (Figs 6I, 7C, G).]	
3	A large basal denticle on the tarsungulum (Fig. 6F: d). No deep median diastema and no chitin-lines on the forcipular coxosternite (Fig. 6F)	***Strigamia* Gray, 1843**
	[In Europe: many species, across most continental lands.]	
–	No large basal denticle on the tarsungulum (Fig. 6G, H). A deep median diastema and distinct chitin-lines on the forcipular coxosternite (Fig. 6G, H: cl)	**4**
4	2 denticles on the mesial side of the tarsungulum (Fig. 6G: dc). Pore-fields absent	***Dignathodon* Meinert, 1870**
	[In Europe: only two species, *D.microcephalus* (Lucas, 1846) (southern Europe; 65–89 LBS) and *D.gracilis* (Attems, 1952) (only recorded in Andalusia; 59–71 LBS.]	
–	No denticles on the mesial side of the tarsungulum (Fig. 6H). Pore-fields present, sub-circular to longitudinally much elongate	***Henia* C.L. Koch, 1847**
	[In Europe: many species, across most continental lands and major islands.]	
5	Coxal pores grouped in 1, 2, 3, or 4 ventral pits, fossae, or distinct clusters on each coxopleuron, all close to the metasternite or covered by it (Fig. 9G–I), but sometimes accompanied by 1 or 2 lateral or posterior separate pores	**6**
	[In France and in north-western Europe, with the exception of *Nothogeophilus* only recorded in England, the labrum of the species of the two other genera present in this area (*Clinopodes* and *Stenotaenia*) generally has numerous bristles and no tubercles (Fig. 7A).]	
–	All coxal pores separate, not in pits, fossae, or distinct clusters (Figs 2E, F, 3H, 9A–F, 10E)	**12**
	[In France and in north-western Europe, the genera included here have one to several tubercles on the intermediate part and bristles on the side pieces of the labrum (Figs 2A, 7B).]	
6	Forcipular coxosternite with a pair of distinctly sclerotised denticles (Fig. 7E)	**7**
–	Forcipular coxosternite without distinctly sclerotised denticles (e.g., Fig. 7G)	**8**
7	Carpophagus structures absent (e.g., Fig. 8G, H). Coxal pores mostly or entirely in 1 ventral pit on each coxopleuron (Fig. 9I)	***Diphyonyx* Bonato, Zapparoli & Minelli, 2008**
	[In Europe: a few species, from the Balkan peninsula to Caucasus. Claws of many anterior legs bearing an enlarged accessory spine.]	
–	Carpophagus structures present (Fig. 8A, B: cs). Coxal pores mostly or entirely in 2–4 ventral pits or clusters (e.g., Fig. 9G)	***Clinopodes* C.L. Koch, 1847**
	[In Europe: many species, across most of central and eastern Europe.]	
8	No pore-fields. Labrum without bristles and without tubercles	***Bebekium* Verhoeff, 1941**
	[In Europe: a single species, *B.mirabile* Verhoeff, 1941; recorded only in the eastern part of the Balkan peninsula; 39–41 LBS.]	
–	Pore-fields present (Fig. 8D, E, G, H: pf). Labrum with bristles, sometimes also with tubercles	**9**
9	All coxal pores in 2 or 3 ventral pits on each coxopleuron (Fig. 9G, H). Pore-fields oval or sub-trapezoid (Fig. 8G: pf)	***Stenotaenia* C.L. Koch, 1847**
	[In Europe: many species, across most of central and eastern Europe.]	
–	All coxal pores in 1 ventral pit on each coxopleuron (Fig. 9I). Pore-fields sub-triangular (e.g., Fig. 8D, E: pf) or reniform or in a transverse band (Fig. 8H: pf)	**10**
10	Carpophagus structures present (Fig. 8E: cs). Pore-fields sub-triangular (e.g., Fig. 8D, E: pf). 57–71 LBS	***Algerophilus* Brolemann, 1925**
	[In Europe: a single species, *Algerophilushispanicus* (Meinert, 1870); only from southern Iberian peninsula.]	
–	Carpophagus structures absent (Fig. 8H). Pore-fields reniform or in a transverse band (Fig. 8H: pf); 37–55 LBS	**11**
11	Pore-fields more or less reniform, ≤ 1/2 of the width of the metasternite; 37–39 LBS	***Nothogeophilus* Lewis, Jones & Keay, 1988**
	[In Europe: a single species, *N.turki* Lewis, Jones & Keay, 1988; only recorded from the isles of Scilly and Wight (southern England). Body length < 15 mm.]	
–	Pore-fields not reniform, > 1/2 of the width of the metasternite (Fig. 8H); 47–55 LBS	***Tuoba* Chamberlin, 1920**
	[In Europe: only two species, *T.poseidonis* (Verhoeff, 1901) (with 49–55 LBS; strictly halophilic, only present on the Mediterranean seashores) and *T.zograffi* (Brolemann, 1900) (47 LBS; not halophilic; only recorded in the Canary islands).]	
12	More than 90 LBS. Pore-fields sub-ovoid/sub-triangular, approx. as long as wide or slightly longer than wide (Fig. 2D)	***Endogeophilus* Bonato, Zapparoli, Drago & Minelli, 2016**
	[In Europe: only 2 species, *E.ichnusae* from Sardinia and *E.alberti* sp. nov. from southern Provence.]	
–	Usually < 90 LBS^1^. Pore-fields not sub-ovoid/sub-triangular (Fig. 8C–F: pf) or absent (Fig. 8B)	**13**
13	Legs of the ultimate pair without an apical claw (Fig. 10A, G, H). Pore-fields either absent (Fig. 8B) or small, sub-circular to slightly elliptical (Fig. 8F: pf)	**14**
–	Legs of the ultimate pair with an apical claw, usually well-developed^2^ (Fig. 10C, D: c, E, F: c, I). Pore-fields usually present^3^, sub-diamond or in a tranverse band (Fig. 8D, E: pf)	**17**
14	Pore-fields present, sub-circular to slightly elliptical (Fig. 8F: pf). Legs of the ultimate pair very elongated, > 2× as long as the penultimate legs (Fig. 10G, H)	***Pleurogeophilus* Verhoeff, 1901**
	[In Europe: at least one species, *P.mediterraneus* (Meinert, 1870); in south-eastern Europe; 61–85 LBS; numerous coxal pores (frequently > 25 on each coxopleuron in adults) and the ultimate metasternite longer than wide (Fig. 9E, F).]	
–	No pore-fields (Fig. 8B). Legs of the ultimate pair usually < 2× as long as the penultimate legs^4^	**15**
15	Forcipular trochanteroprefemur ~ as long as wide and without denticle (Fig. 7F: tp). Metasternite of the ultimate leg-bearing segment wider than long (Fig. 9B)	***Galliophilus* Ribaut & Brolemann, 1927**
	[In Europe: only one species, *G.beatensis*, recorded confidently only in the eastern Pyrenees; ~ 81–85 LBS; tarsungulum sharply compressed in the internal side, making almost a right angle in horizontal view (Fig. 7F).]	
–	Forcipular trochanteroprefemur much longer than wide and with a denticle (Figs 6I, 7C: tp). Metasternite of the ultimate leg-bearing segment longer than wide (Fig. 9A)	**16**
16	39–41 LBS. Forcipular coxosternite slightly wider than long. All forcipular articles with a denticle (Fig. 7C)	***Arctogeophilus* Attems, 1909**
	[In Europe: three species, *A.inopinatus* (Ribaut, 1911) (western and central France), *A.attemsi* Folkmanová, 1956, and *A.macrocephalus* Folkmanova & Dobroruka, 1960 (both only recorded in Ukraine).]	
–	67–87 LBS. Forcipular coxosternite slightly longer than wide. Forcipular trochanteroprefemur and tarsungulum with a denticle, intermediate forcipular articles without denticle (Fig. 6I)	***Gnathoribautia* Brolemann, 1909**
	[In Europe: only 2 species, *G.bonensis* (67–83 LBS; body length ≤ 7 cm; Macaronesia, Iberian peninsula, and Sicily) and *G.syriaca* (Attems, 1903) (~ 87 LBS; body length ≤ 11 cm; Aegean islands]	
17	Metasternite of the ultimate leg-bearing segment longer than wide (Figs 9D, 10I)	**18**
–	Metasternite of the ultimate leg-bearing segment wider than long (Fig. 9C)	**19**
18	Forcipular trochanteroprefemur and tarsungulum each with a denticle. Legs of the ultimate leg-bearing segment without elongate projections (Fig. 10F)	***Pachymerium* C. L. Koch, 1847**
	[In Europe: few species, across most of Europe. Numerous coxal pores, > 20 widely distributed on each coxopleuron in adults (Fig. 9D).]	
–	Forcipules without denticles. Legs of the ultimate leg-bearing segment with elongate projections (Fig. 10I)	***Acanthogeophilus* Minelli, 1982**
	[In Europe: only 1 species, *A.dentifer* Minelli, 1982, only recorded in the Italian peninsula; ~ 67 LBS.]	
19	Forcipular coxosternite > 2× as wide as long (Fig. 7D). Tarsungulum strongly narrowing near the base, very narrow for most of its length and distinctly compressed also along the external side (Fig. 7D)	***Eurygeophilus* Verhoeff, 1899**
	[In Europe: only 2 species, *E.pinguis* (Brolemann, 1898) (< 50 LBS) and *E.multistiliger* (Verhoeff, 1899) (> 50 LBS). Pore-fields in a very stretched transverse band in both species (Fig. 8C: pf).]	
–	Forcipular coxosternite < 2× as wide as long (Fig. 7G; also F). Tarsungulum gradually narrowing and compressed only along the internal side (Fig. 7G)	***Geophilus* Leach, 1814**
	[In Europe: many species. With the exception of *G.carpophagus* Leach, 1815, which has pore-fields in a very stretched transverse band, the other species have either stretched sub-triangular/sub-diamond pore-fields (Fig. 8D, E: pf) or no pore-fields^3^.]	

**Figure 6.**
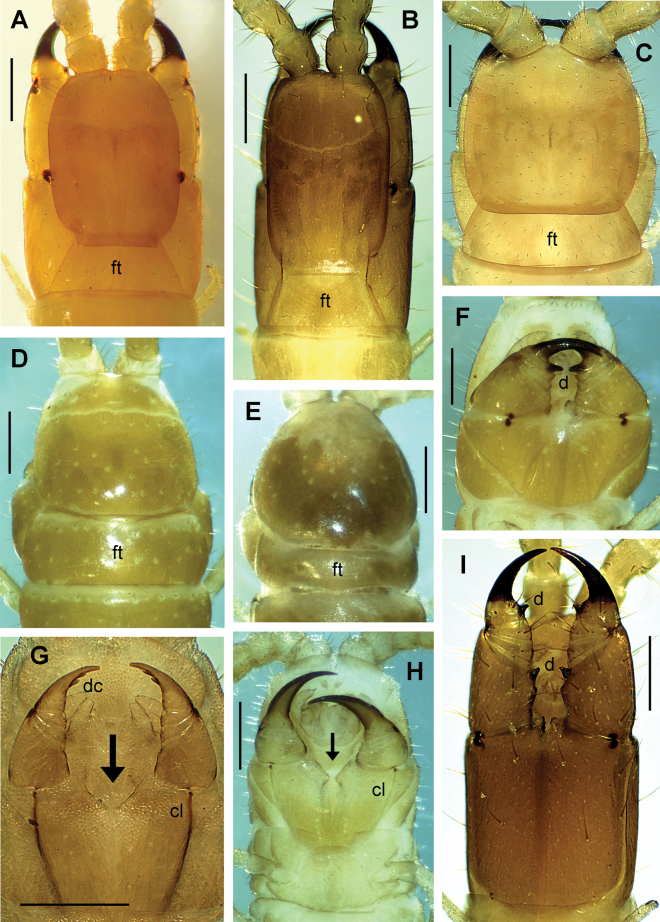
Head and forcipular segment. Species **A***Pachymeriumferrugineum***B***Gnathoribautiabonensis***C***Pleurogeophilusmediterraneus***D***Strigamiacarniolensis***E***Heniavesuviana***F***Strigamiacarniolensis***G***Dignathodonmicrocephalus***H***Heniabicarinata***I***Gnathoribautiabonensis*. Views: dorsal (**A–E**), ventral (**F–I**). Abbreviations: cl chitin-lines, d forcipular denticle, dc denticles of the mesal side of the forcipular tarsungulum, ft forcipular tergite. Arrow: median diastema of the forcipular coxosternite. Scale bars: 400 µm (**A–F, H-I**); 300 µm (**G**).

**Figure 7.**
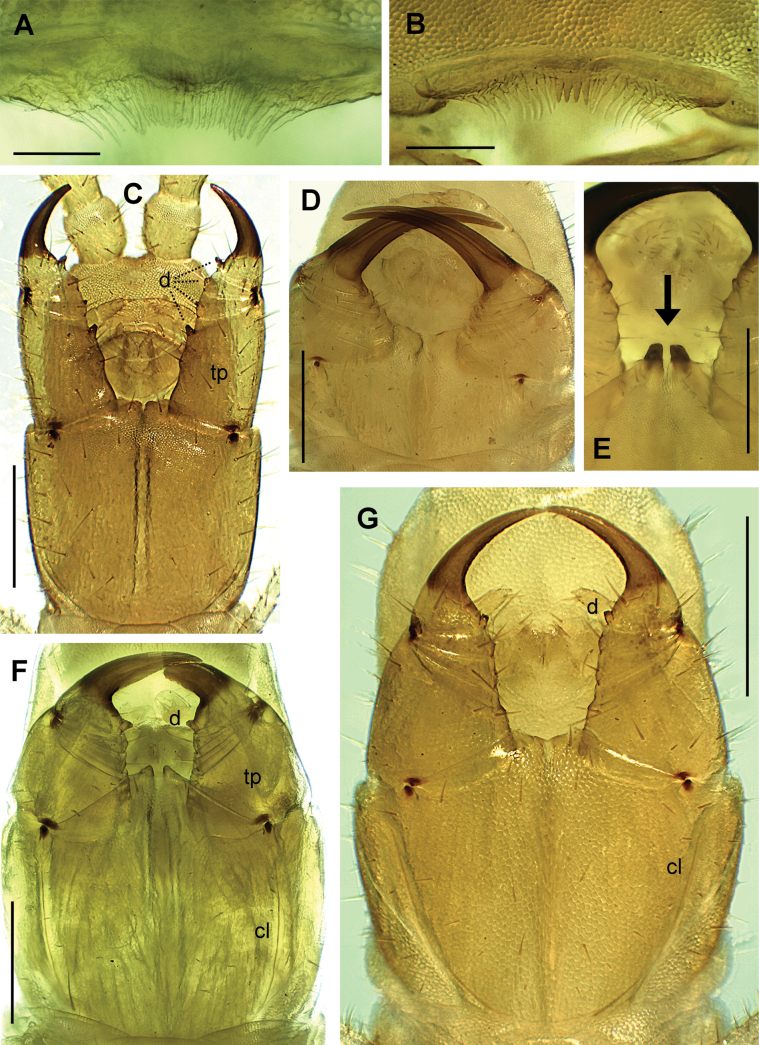
**A, B** labrum **C, D, F, G** forcipular segment **E** distal part of the forcipular coxosternite. Species **A***Clinopodesvesubiensis***B***Geophilusstuderi***C***Arctogeophilusinopinatus***D***Eurygeophiluspinguis***E***Clinopodesvesubiensis***F***Galliophilusbeatensis***G***Geophilusgavoyi*. Views: all ventral. Abbreviations: cl chitin-lines, d forcipular denticle, tp trochanteroprefemur. Arrow: denticles on the forcipular coxosternite. Scale bars: 50 µm (**A, B**); 300 µm (**C–G**).

**Figure 8.**
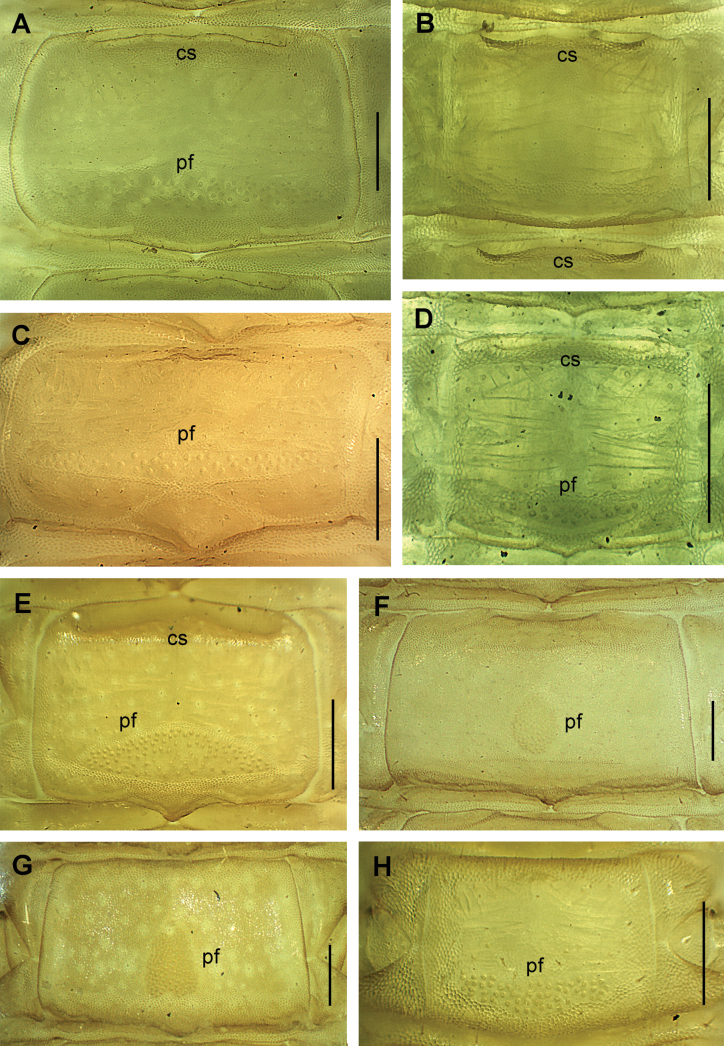
Metasternite of an anterior LBS. Species **A***Clinopodesvesubiensis*LBS 16 **B***Galliophilusbeatensis*LBS 19 **C***Eurygeophiluspinguis*LBS 10 **D***Geophilusosquidatum*LBS 11 **E***Geophiluselectricus*LBS 11 **F***Pleurogeophilusmediterraneus*LBS 15 **G***Stenotaenialinearis*LBS 20 **H***Tuobaposeidonis*LBS 12. All ventral views. Abbreviations: cs carpophagus-structure, pf pore-field. Scale bars: 200 µm.

**Figure 9.**
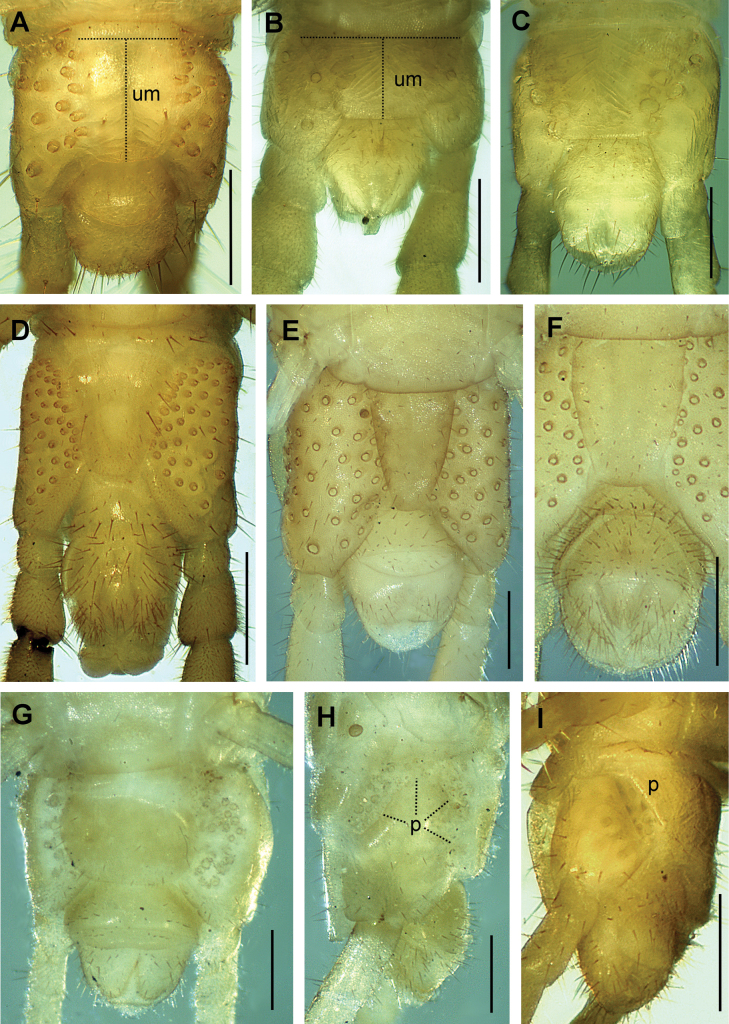
Ultimate LBS without distal part of legs. Species **A***Arctogeophilusinopinatus* female **B***Galliophilusbeatensis* male **C***Geophilusflavus* female **D***Pachymeriumferrugineum* male **E***Pleurogeophilusmediterraneus* female **F***Pleurogeophilusmediterraneus* male **G**, **H***Stenotaenialinearis* female **I***Tuobaposeidonis* female. Views: ventral (**A–G**), lateral (**H, I**). Abbreviations: p pit or fossa (**H** wide fossa from dorsal to ventral side, indicated by dotted lines), um metasternite of the ultimate LBS (medial length and maximum width are indicated by dotted lines). Scale bars: 300 µm.

**Figure 10.**
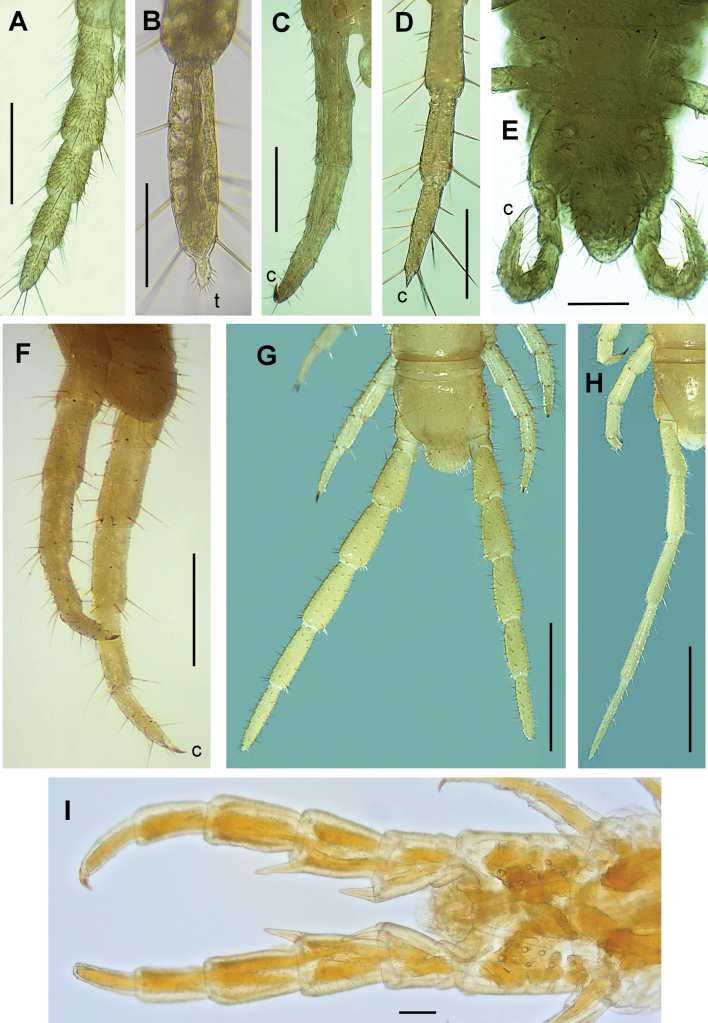
Legs of the ultimate pair **A, C** right leg **B, D** distal articles of the right leg **E–G** both legs **H** left leg. Species **A***Arctogeophilusinopinatus* male **B***Arenophilusperegrinus* female **C***Eurygeophiluspinguis* female **D***Geophilusfucorum* female **E***Geophilusrichardi* female **F***Pachymeriumferrugineum* female **G***Pleurogeophilusmediterraneus* male **H***Pleurogeophilusmediterraneus* female **I***Acanthogeophilusspiniger* female. Views: ventral (**A-E, I**), lateral (**F**) and dorsal (**G-H**). Abbreviations: c claw, t tubercle with spines. Scale bars: 300 µm (**A, C, F**); 50 µm (**B**); 100 µm (**D, E, I**); 1 mm (**G–H**).

## ﻿Discussion

### ﻿Taxonomic remarks

After the discovery of a specimen of *Endogeophilus* among the centipedes collected in the Port-Cros National Park, further sessions of field research were conducted with the aim to find other specimens, either in the collection locality or other sites. However, efforts have remained ineffective up to now. Worth notice is that the only three specimens of *Endogeophilus* previously reported were found through intense field research and among a huge sample of collected centipedes (Bonato et al. 2016), and no other specimens have been found subsequently.

We observed several morphological differences between the single individual of *Endogeophilus* collected in Provence and the three individuals previously collected in Sardinia (Table 1). However, only some differences may be attributed confidently to evolutionary divergence (thus suggesting that they represent different species), while other differences may be explained by variability between individuals (including variation associated to body size or sex). Moreover, the effect of “errors” in measurement and character evaluation may not be negligible in such a small sample. The very few specimens available for comparison did not allow testing these alternative explanations empirically or statistically. However, we tentatively tabulate what is known for other better-studied geophilids (Table 1). Following a cautionary approach, at least a few differences are very difficult to explain by intraspecific variation or measurement errors alone. Thus, these differences indicate an evolutionary morphological divergence between the population of *Endogeophilus* discovered in Provence (represented by the single specimen here described) and the populations inhabiting south-western Sardinia (the three specimens described as *E.ichnusae*). These differences are in the shape of the pretarsi of the second maxillae and the forcipules.

The maxillary pretarsi are more slender and distinctly hooked in the specimen from Provence, while they are stouter and not hooked in all three Sardinian specimens (Fig. 3D vs Fig. 4B). There is no asymmetry between right and left pretarsi. The observed difference is larger than the interindividual variation observed within well-studied species of geophilids (unpublished data), and the effect of body size may be ruled out considering the close lengths of the specimens (Table 1).

The forcipules of the specimen from Provence differ from those of all three Sardinian specimens because they are slightly more elongate in comparison with the coxosternite, with stouter trochanteroprefemora and more slender tarsungula, which are also more gradually narrowing (Fig. 3E, F vs Fig. 4A, B). There is no asymmetry between right and left forcipules, and no differences between sexes among the Sardinian specimens. The observed differences are larger than usually observed in other geophilids (unpublished data), even in comparison to changes observed or expected with growth (Table 1).

Other observed differences may turn out to be variable characters between the two species, but further specimens are necessary to rule out alternative explanations (Table 1). These putative characters include the length of the setae, the relative size of the first maxillary lappets, the elongation of the forcipular metatergite, the depth of the carpophagus pits, the range of variation of the number of legs, the number of coxal pores in relation to the body size, and the relative length of the legs of the ultimate pair.

### ﻿Identification key to genera of Geophilidae

The redefinition of the diagnosis of *Endogeophilus* prompted us to build a key to all geophilid genera recorded from Europe thus far, according to the current taxonomy and nomenclature in use, and leveraging all recently published information on the morphology of European geophilids. Presently, the Geophilidae s.l. living in Europe (~ 120 species; Bonato et al. 2014a) are recognised in 20 genera (Table 2).

For a long time, the identification of geophilids collected in Europe have relied on consulting a few specialists with personal expertise, browsing descriptions that are sparse in the taxonomic literature (often heterogeneous and only partially comparable) or by using a few available keys. After the keys published by Attems (1929) within his global monograph on Geophilomorpha (now largely outdated), more recent keys (still suitable for European geophilids) have been published to cover single countries or regions (e.g., Koren 1986; Andersson et al. 2005; Barber 2009; Dányi 2010; Iorio and Labroche 2015; Iorio et al. 2022b). Indeed, an interactive digital key to all European species of Geophilomorpha has been recently delivered on-line (Bonato et al. 2014a); however, it includes many characters often requiring anatomical dissection, and most of the characters are not accompanied by illustrations. Given the current state of uncertainty of the species-level taxonomy of many European genera of geophilids (e.g., Del Latte et al. 2015; Bonato et al. 2023), a simplified tool assisting in distinguishing genera may turn out to be useful and desirable for many faunistic and ecological investigations. Moreover, we have given priority to characters that are easier to evaluate (not requiring high magnification, dissection, or clarification of the integument) and of broader applicability (effective for adult and large immature individuals, and without knowing the sex of the specimen).

### ﻿Biogeographic and conservation remarks

The new species of *Endogeophilus* has been discovered in southern Provence, ca 2 km from the coast, while the known range of *E.ichnusae* is limited to south-western Sardinia (Fig. 1). The two areas are > 450 km far apart, and are separated by a broad branch of sea. Similar distributional disjunctions of strictly related species between Sardinia and Provence are apparently rare among soil arthropods, even though single species have been reported with separate populations in Sardinia and narrow parts of continental Europe (e.g., Soldati and Soldati 2014; Ponel et al. 2023). Among centipedes, for instance, the himantariid *Stigmatogastersardoa* (Verhoeff, 1901) has been recently recorded in Provence while it was previously recorded only in Sardinia (ÉI, pers. obs.).

Provence is one of the most intensely surveyed areas in Europe for centipedes: in June 2024, ~ 6,100 records and 12,300 identified specimens had been collected for the entire Provence-Alpes-Côte d’Azur region (Myria-France 2024; Iorio and Racine, unpublished data). However, even within Provence, several sectors and/or habitats remain poorly studied, like the south of the Var department and generally the strictly endogeic centipedes. In particular, *E.alberti* has been found in the peripheric area (“zone d’adhésion”) of the Port-Cros National Park, which — like the massif of the Maures — was poorly studied before 2022, except for coastal habitats (Iorio 2014, 2022; Iorio et al. 2020, 2023; Myria-France 2024).

Because the Alpes-Maritimes department, as well as secondarily the northern part of the Var department, have been relatively well studied, it seems possible that *E.alberti* sp. nov. has a narrow distribution in Provence, possibly limited to the massif of the Maures and its surroundings. However, as only one specimen was found after > 50 hours dedicated to its search and the single specimen corresponds to only 0.6% of the total number of collected geophilomorphs, this species shows a very low probability of detection in comparison with most other centipedes.

While Provence belongs to a well-known biodiversity hotspot within Europe (Médail and Quézel 1997), its biodiversity is highly threatened by the demographic pressure and various human impacts (e.g., urbanisation, artificialisation of natural environments, fires) (Daligaux 2003; Médail and Diadema 2006; Vimal et al. 2012). In particular, in the last decades, the forest habitats of the south of the Maures have been disturbed by urbanisation and artificialisation. Also, the fires caused a strong impact in this area, as in 2021 with 7,000 hectares burned (Ballouard et al. 2023). These impacts also concern several species of centipedes (Chilopoda), which are already considered as threatened (Iorio et al. 2015, 2020, 2023; Geoffroy and Iorio 2019; Iorio 2022; Iorio et al. 2022a).

Further studies would be necessary to precisely define the real distribution and abundance of *E.alberti*, its ecology, and its conservation status.

## Supplementary Material

XML Treatment for Endogeophilus

XML Treatment for Endogeophilus alberti

## Footnotes

Apart from Endogeophilus, only one species of European geophilids has > 90 LBS: Stenotaenia sturanyi (Attems, 1903) (105–115 LBS), recorded between Macedonia and the Chalcidic peninsula, possibly also in Crete (Bonato and Minelli 2008).

European species of Geophilus with poorly developed claws on the ultimate legs of both sexes include G. fucorum, G. joyeuxi Léger & Duboscq, 1903 (variable), and G. seurati Brolemann, 1924. Other species may have poorly developed claws on the ultimate legs of males only, e.g., G. flavus (variable) and G. carpophagus Leach, 1815.

A few European species of Geophilus have no pore-fields: G. guanophilus Verhoeff, 1939, G. ribauti Brolemann, 1908, G. richardi Brolemann, 1904, G. truncorum Bergsøe & Meinert, 1866, and other species of uncertain identity (G. madeirae Latzel, 1895, G. ibericus (Attems, 1952), G. strictus Latzel, 1880).

Gnathoribautia bonensis has a fairly elongated ultimate pair of legs, but generally less so than Pleurogeophilus mediterraneus. Also, G. bonensis has a much longer head than P. mediterraneus (Fig. 6B, C) and distinct forcipules (Fig. 6I).

## ﻿Additional information

### Author ORCIDs

Étienne Iorio https://orcid.org/0009-0003-2550-3480

Lucio Bonato https://orcid.org/0000-0002-8312-7570

### Data availability

All of the data that support the findings of this study are available in the main text.
